# Multigenotype Q Fever Outbreak, the Netherlands

**DOI:** 10.3201/eid1504.081612

**Published:** 2009-04

**Authors:** Corné H.W. Klaassen, Marrigje H. Nabuurs-Franssen, Jeroen J.H.C. Tilburg, Maurice A.W.M. Hamans, Alphons M. Horrevorts

**Affiliations:** Canisius Wilhelmina Hospital, Nijmegen, the Netherlands (C.H.W. Klaassen, M.H. Nabuurs-Franssen, J.J.H.C. Tilburg, A.M. Horrevorts); Radboud University Medical Centre, Nijmegen (M.H. Nabuurs-Franssen); Food and Consumer Product Safety Authority, Eindhoven, the Netherlands (M.A.W.M. Hamans)

**Keywords:** Coxiella burnetii, DNA fingerprinting, epidemiology, outbreak, letter

**To the Editor:** Q fever is a zoonosis caused by *Coxiella burnetii* (*1*). An ongoing Q fever outbreak has occurred in the Netherlands since 2007; incidence rates have increased >50-fold compared with the baseline rate (*2*). The source of this outbreak is unknown. Identifying the source of an infection is complicated because of difficulties in obtaining sufficient clinical and/or environmental samples for testing. Molecular diagnosis of Q fever has focused on the use of serum samples. Up-to-date genotyping of *C. burnetii* has depended on cultivation and enrichment of the isolate before analysis (*3*). We report multiple-locus variable-number tandem repeat analysis (MLVA) typing of *C. burnetii* for a variety of human and animal clinical samples obtained from different locations in the Netherlands (Table).

**Table:** Genotyping results for human and animal clinical samples, Q fever outbreak, the Netherlands

Patient/animal no.	Sample	Ms27*	Ms28*	Ms34 *	Symptoms	Ct value†	Location
Patient 1	Plasma	3	3	8	Severe	34.4	1
Ewe 1	Vaginal swab	3	3	8	None	25.7	1
Ewe 2	Vaginal swab	3	3	8	None	16.3	1
Ewe 3	Vaginal swab	3	3	8	None	18.8	1
Lamb 1	Throat swab	3	3	8	None	27.9	1
Lamb 2	Throat swab	3	3	8	None	29.9	1
Lamb 3	Throat swab	3	3	8	None	28.9	1
Patient 2	Urine	3	3	7	None	31.7	2
	Throat swab	3	3	7		31.8	
Patient 3	Urine	NR‡	3	4	Mild	36.7	2
Patient 4	Sputum	4	3	7	Severe	34.2	2
Patient 5	Sputum	3	3	7	Severe	31.9	3
Nine Mile	Reference strain	4*	6*	5*			

*The allele-calling convention used was as published (*3*), resulting in a 4, 6, 5 code assigned respectively to the 6-bp repeat unit loci Ms-27, Ms-28, and Ms-34 for the genome sequence of the Nine Mile RSA-493 strain (GenBank accession no. NC002971.1). Primers for these markers were redesigned to amplify significantly shorter PCR products and were combined into 1 multicolor multiplex PCR. Primer sequences for Ms-27 were 5’-HEX-TCTTTATTTCAGGCCGGAGT-3’ and 5’-GAACGACTCATTGAACACACG-3;for Ms-28, 5’-TMR-AGCAAAGAAATGTGAGGATCG-3’and 5’- GCCAAAGGGATATTTTTGTCCTTC-3’; for Ms-34, 5’-FAM-TTCTTCGGTGAGTTGCTGTG-3’ and 5’-GCAATGACTATCAGCGACTCGAA-3’. †Cycle threshold (Ct) value obtained by using real-time PCR targeting the IS1111a element. ‡NR, no result obtained. A full genotype was obtained only in samples with the highest DNA loads (Ct value <35).

Severe pneumonia developed in patient 1 after close contact with sheep (ewes) and intimate cuddling with a newborn lamb. Patients 2 and 3 (a dairy goat farmer and his wife from another village) tested positive for Q fever after a large part of their goat herd aborted offspring. The farmer had no clinical symptoms; his wife had mild symptoms that disappeared spontaneously within 2 days. No samples from any of the goats were available. Two additional patients were tested, 1 of which lived in the same village as patients 2 and 3.

Swab specimens from all sheep and lambs tested in the first case yielded identical MLVA genotypes. The same genotype was also found in patient 1 but not in the other examined samples, implicating sheep as the origin of patient 1’s infection. Although patients 2 and 3 live together, the genotype found in patient 2 differed from the (partial) genotype found in patient 3. Yet another genotype was found in a patient from the same village (patient 4). However, an identical genotype found in patient 2 was found in a patient from a distant village (patient 5). The village had only 1 goat farm, and if this herd of goats was the source of infection for the farmer, his wife, and patient 4, it would have contained >1 genotype. At least 1 of the obtained genotypes has spread over a wider surface area in the Netherlands.

Our results show that the unprecedented, ongoing Q fever outbreak in the Netherlands involves multiple genotypes of *C. burnetii*. Because most of the genotypes differ only by a single repeat difference, they might represent microvariants of a hypervirulent strain that has been introduced in the Dutch animal population. MLVA schemes with up to 17 markers have been previously reported (*3*). In this “proof of concept” (applying direct genotyping of *C. burnetti* on clinical samples), we focused on the 3 shortest repeat units because we believed that these units might have the highest à priori chance of successful amplification in clinical samples (especially in serum/plasma). Similar genotypes as those reported here were found in the MLVA database (http://mlva.u-psud.fr), but these similarities need confirmation by using more markers. Although using only 3 markers may lead to poor discriminatory power, we were still able to distinguish 4 different genotypes in a relatively small collection of serum samples. We are currently exploring the use of additional MLVA markers.

Our results also show a poor correlation between DNA load and clinical symptoms. Multiple human and animal clinical samples, including serum and plasma, throat or genital swabs, or sputum and urine, may be useful for direct genotyping and outbreak source tracking.
